# Protect Effects of Perilla Seed Extract and Its Active Ingredient Luteolin Against Inflammatory Bowel Disease Model via the PI3K/AKT Signal Pathway In Vivo and In Vitro

**DOI:** 10.3390/ijms26083564

**Published:** 2025-04-10

**Authors:** Jin Zhang, Linlu Zhao, Jieyi He, Huining Wu, Mengru Guo, Zhichao Yu, Xingbin Ma, Yanhong Yong, Youquan Li, Xianghong Ju, Xiaoxi Liu

**Affiliations:** Department of Veterinary Medicine, College of Coastal Agricultural Sciences, Guangdong Ocean University, Zhanjiang 524091, China; zhangjin30@stu.gdou.edu.cn (J.Z.);

**Keywords:** perilla seed extract, network pharmacology, transcriptomics analysis, IBD, luteolin

## Abstract

The purpose of this study was to investigate the anti-inflammatory effects of Perilla Seed Extract (PSE) and its active ingredient on Inflammatory Bowel Disease (IBD) in vitro and in vivo. Thirty-two C57/BL mice were randomly divided into four groups (*n* = 8): control group (CON), PBS group, LPS group (LPS 3.5 mg/kg given intraperitoneally [ip] on day 7 of the study only), and PSE group (100 mg/kg orally daily + LPS ip at 3.5 mg/kg on day 7). Mice were euthanized 24 h after LPS administration. MODE-K cells were divided into five groups: control group (CON), LPS group (50 μg/mL LPS for 2 h), and PSE group (low dose, 25 μg/mL PSE + LPS; middle dose, 50 μg/mL PSE + LPS; high dose, 100 μg/mL PSE + LPS). In vivo, compared with the CON group, LPS revealed a significant decrease in the villus length-to-crypt depth ratio (*p* < 0.01) and goblet cell density per unit area (*p* < 0.01). Conversely, PSE administration resulted in a significant increase in the villus length-to-crypt depth ratio (*p* < 0.01) and goblet cell density (*p* < 0.01). LPS significantly increased the ROS content (*p* < 0.01), the secretion of inflammatory cytokines of IL-6 (*p* < 0.01), TNF-α (*p* < 0.01), and the mRNA expressions of *HO-1* (*p* < 0.01). LPS significantly decreased the mRNA expressions of *Occludin* (*p* < 0.01) and *Claudin1* (*p* < 0.01). In contrast, PSE treatment led to a marked decrease in ROS levels (*p* < 0.01), along with a reduction in the secretion of inflammatory factors IL-6 (*p* < 0.01) and TNF-α(*p* < 0.05), as well as the mRNA expressions of *HO-1* (*p* < 0.01). Concurrently, PSE significantly increased the mRNA expressions of *Occludin* (*p* < 0.05) and *Claudin1* (*p* < 0.01). In vitro, PSE treatment also significantly reversed LPS-induced inflammation, oxidation and tight junction–related factors. Network pharmacology identified 97 potential targets for PSE in treating IBD, while transcriptomics analysis revealed 342 differentially expressed genes (DEGs). Network pharmacology and transcriptomics analysis indicated that significant pathways included the PI3K-Akt signaling pathway, MAPK signaling pathway, and TNF signaling pathway, of which the PI3K-AKT pathway may represent the primary mechanism. In an in vivo setting, compared with the CON group, LPS led to a significant increase in the protein expression of p-PI3K/PI3K (*p* < 0.01) and p-AKT1/AKT1 (*p* < 0.01). Conversely, PSE resulted in a significant decrease in the protein expression of p-PI3K/PI3K (*p* < 0.01) and p-AKT1/AKT1 (*p* < 0.01). In vitro, compared with the LPS group, PSE also significantly blocked the protein expression of p-PI3K/PI3K (*p* < 0.01) and p-AKT1/AKT1 (*p* < 0.01). The chemical composition of PSE was analyzed using UPLC-MS/MS, which identified six components including luteolin (content 0.41%), rosmarinic acid (content 0.27%), α-linolenic acid (content 1.2%), and oleic acid (content 0.2%). Molecular docking found that luteolin could establish stable binding with eight targets, and luteolin significantly decreased the p-AKT1/AKT1 ratio (*p* < 0.01) compared to the LPS group in MODE-K cells. In summary, PSE demonstrates efficacy against IBD progression by enhancing intestinal barrier function and inhibiting inflammatory responses and oxidative stress via the PI3K/AKT signaling pathway, and luteolin’s inhibition of AKT1 protein phosphorylation appears to play a particularly crucial role in this therapeutic mechanism.

## 1. Introduction

Inflammatory bowel disease (IBD) is a chronic intestinal disorder encompassing ulcerative colitis (UC) and Crohn’s disease (CD) [1,2]. Although observed in both humans and animals, the exact pathogenesis of IBD remains elusive [3]. The clinical manifestations of IBD include depression, loss of appetite, abdominal pain, diarrhea, weight loss, and intestinal bleeding, among other symptoms [4,5]. In China, the increasing scale of livestock breeding has been accompanied by a rise in IBD incidence, leading to significant economic losses in the livestock industry. Currently, antibiotics and biological agents are the primary therapeutic interventions for managing IBD in animal production. However, these approaches face challenges, including the presence of drug residues and potential adverse effects [6,7]. The development of safe and effective therapeutic agents remains a critical and challenging area in IBD research. In July 2020, the Ministry of Agriculture and Rural Affairs of the People’s Republic of China issued Announcement No. 194, mandating that feed production enterprises cease the use of growth-promoting drug feed additives, with the exception of traditional Chinese medicine [8]. Due to their safety profile and minimal side effects, Chinese herbal medicine extracts, which are rich in active ingredients, have garnered increasing attention as feed additives to regulate intestinal health and promote growth [9].

Network pharmacology can construct a network model based on the complex relationship between diseases, active components, therapeutic targets, and signaling pathways of traditional Chinese medicine. A combination of computational and experimental methods is employed to identify drug targets, predict drug efficacy and potential side effects, and design safer and more effective therapeutic interventions [10]. Validation of network pharmacology findings can involve molecular docking, and can be integrated with transcriptomics analysis to ascertain the active constituents and molecular pathways of Chinese herbal medicines. Molecular docking simulates the binding interactions between bioactive components and target proteins, whiletranscriptomics analysis evaluates differentially expressed genes (DEGs) in individuals [11,12].

*Perilla frutescens*, a traditional medicinal plant widely cultivated in Asia, has a long history of use for both medicinal and culinary purposes in China [13]. Presently, the predominant application of perilla seeds is in the production of perilla seed oil (PSO), which is rich in unsaturated fatty acids such as alpha-linolenic acid (ALA) and exhibits various biological activities such as antioxidant, anti-inflammatory, and immunomodulatory activities, among others [14,15]. PSE has been demonstrated to exert a preventative effect on aberrant colonic epithelial cell progression and has a preventative potential for use in the management of IBD in a murine model [16,17]. However, further research is required to elucidate the precise mechanisms by which PSE and its active ingredients exert their anti-inflammatory effects. Consequently, there is a necessity to identify more active substances with high efficiency from the active ingredients of PSE except ALA. To resolve the aforementioned issues, network pharmacology, molecular docking, transcriptomics analysis, and real experimental verification were integrated to address this question, providing a theoretical foundation for future research and therapeutic strategies targeting IBD.

## 2. Results

### 2.1. The Effect of PSE on LPS-Induced IBD in Mice

#### 2.1.1. Effect of PSE on Blood Indices of Mice

**Figure 1:**
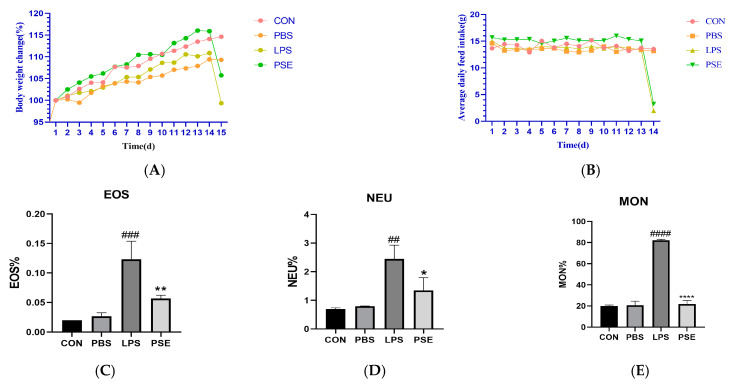
Effect of PSE in mice administered with LPS to induce enteritis. The experimental groups were divided into four groups: (i) CON (drinking water), (ii) PBS (PBS), (iii) LPS (3. 5mg/kg BW), (iv) PSE (3.5 mg/kg and PSE 100 mg/kg/d) *n* = 8. Effect of PSE on % change in average body weight (**A**), Effect of PSE on daily feed intake (**B**), and eosinophils (**C**), neutrophils (**D**), peripheral blood monocytes (**E**). PSE can alleviate body weight loss caused by LPS and has anti-inflammatory effects. Data are expressed as mean ± SEM. ## *p* < 0.01, ### *p* < 0.001, and #### *p* < 0.0001 vs. control group; * *p* < 0.05, ** *p* < 0.01, and **** *p* < 0.0001 vs. LPS control group.

Body weight (BW) and daily feed intake were monitored throughout the study period (Figure 1A,B). Mice in the PSE group exhibited higher average body weights compared to both CON and LPS groups from days 7 to 14. On day 14, following intraperitoneal LPS injection in the LPS and PSE groups, both feed intake and average body weight decreased markedly within 24 h (Figure 1A,B). Post-sacrifice analysis revealed that LPS administration significantly increased the percentage of eosinophils (EOS%, *p* < 0.001), neutrophils (NEU%, *p* < 0.01), and monocytes (MON%, *p* < 0.001) in peripheral blood, indicating systemic inflammation. PSE treatment significantly reduced EOS% (*p* < 0.01), NEU% (*p* < 0.05), and MON% (*p* < 0.001) compared to the LPS group (Figure 1C–E), demonstrating its anti-inflammatory effects.

#### 2.1.2. Effects of PSE on Organ Index and Jejunal Histopathology in LPS-Induced Colitis Mice

**Figure 2:**
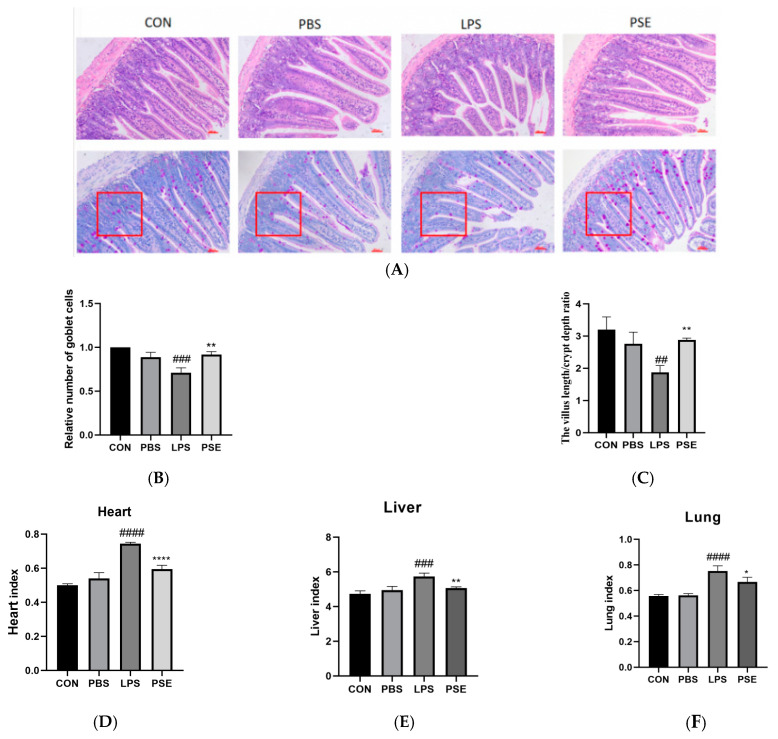
Protective effect of PSE on UC mice. The experimental groups were divided into four groups: (i) CON (drinking water), (ii) PBS (PBS), (iii) LPS (3.5 mg/kg BW), (iv) PSE (3.5 mg/kg and PSE 100 mg/kg/d) *n* = 8. H&E and PAS staining The image above is a slice of the mouse jejunum observed (200×). The red box represents the unit area of counting goblet cell numbers. Scale bar represents 100 μm (**A**), and the relative number of goblet cells (**B**), the villus length/crypt depth ratio (**C**), organ index (**D**–**F**). PSE treatment effectively ameliorated LPS-induced intestinal and organ damage. Data are expressed as mean ± SEM. ## *p* < 0.01, ### *p* < 0.001, #### *p* < 0.0001 vs. control group; * *p* < 0.05, ** *p* < 0.01, **** *p* < 0.0001 vs. LPS control group.

Histological examination of the CON group showed normal intestinal structure, while the LPS group exhibited significant jejunal damage with marked villous loss. Compared with the CON group, the LPS group showed a significant decrease in both the villus length-to-crypt depth ratio (Figure 2A) and goblet cell density per unit area (Figure 2C). PSE administration resulted in significant improvement of jejunal structure, evidenced by an increased villus length-to-crypt depth ratio (*p* < 0.01, Figure 2B) and elevated goblet cell density (*p* < 0.01, Figure 2C). LPS treatment significantly increased heart, liver, and lung indices compared to the CON group. The PSE group exhibited significantly reduced heart, liver, and lung indices compared to the LPS group (Figure 2D–F), indicating that PSE treatment effectively ameliorated LPS-induced intestinal and organ damage.

#### 2.1.3. Effects of PSE on Inflammation, Oxidation, and Tight Junction–Related Factors in LPS-Induced Colitis Mice

**Figure 3:**
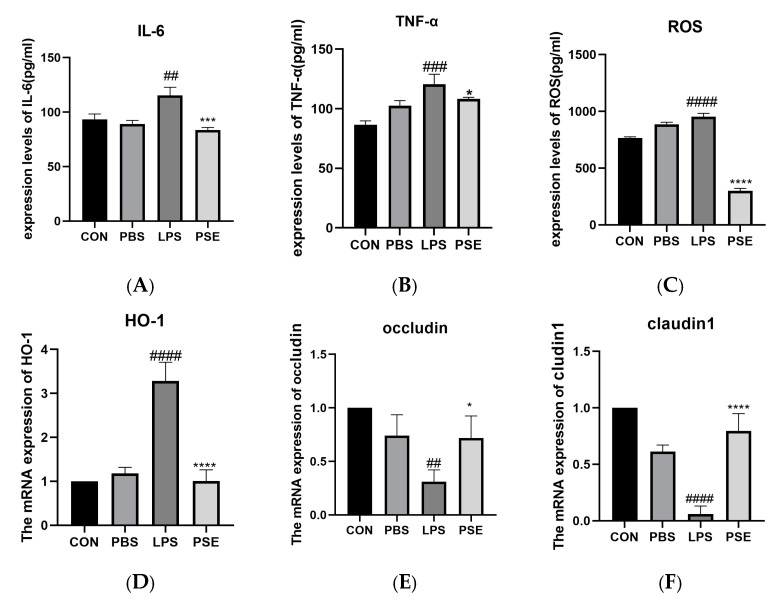
The effects of PSE on inflammatory factors, oxidation indexes, and tight junction factors in the LPS-induced colitis mice model. The experimental groups were divided into four groups: (i) CON (drinking water), (ii) PBS (PBS), (iii) LPS (3.5 mg/kg BW), (iv) PSE (3.5 mg/kg and PSE 100 mg/kg/d). *n* = 8. Inflammatory factors IL-6 and TNF-α (**A**,**B**), oxidation indexes ROS and HO-1 (**C**,**D**), tight junction factors Occludin and Claudin1 (**E**,**F**). PSE effectively inhibited the expression of inflammatory and oxidative factors and increased the expression of tight junction factors. Data are expressed as mean ± SEM. ## *p* < 0.01, ### *p* < 0.001, #### *p* < 0.0001 vs. control group; * *p* < 0.05,*** *p* < 0.001, **** *p* < 0.0001 vs. LPS control group.

LPS treatment significantly increased the secretion of inflammatory cytokines IL-6 (*p* < 0.01) and TNF-α (*p* < 0.001), elevated ROS content, and enhanced messenger RNA(mRNA)expression of the antioxidative enzyme HO-1 in the jejunum compared to the CON group, indicating severe inflammatory response and oxidative stress. PSE treatment significantly reduced IL-6 (*p* < 0.001) and TNF-α (*p* < 0.05) secretion, decreased ROS content (*p* < 0.001), and attenuated HO-1 mRNA expression compared to the LPS group, demonstrating anti-inflammatory and antioxidative effects. Additionally, LPS significantly decreased the mRNA expression of tight junction proteins Occludin (*p* < 0.01) and Claudin1 (*p* < 0.001). PSE treatment significantly increased the mRNA expression of both Occludin (*p* < 0.01) and Claudin1 (Figure 3E,F).

### 2.2. Network Pharmacology Analysis of Potential Targets for PSE in IBD

**Figure 4:**
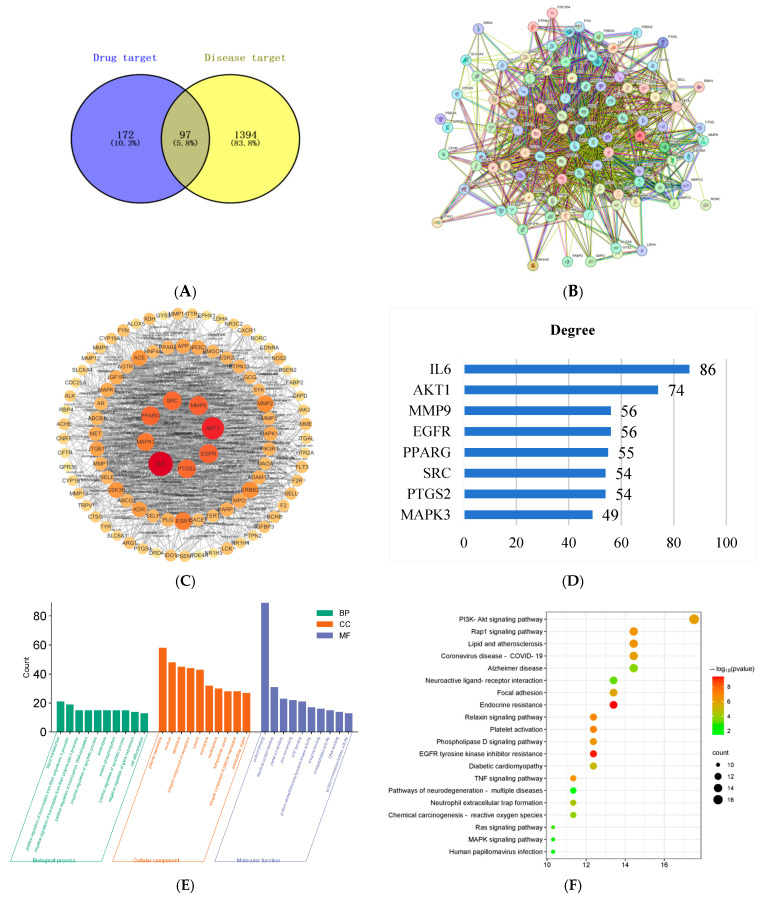
Network pharmacology analysis. Venn diagram (**A**). PPI network of intersecting genes (**B**). Visualization of PPI network (**C**). The core target for the top eight of the Degree value (**D**). Go analysis (**E**). KEGG pathway analysis (**F**).

The Swiss Target Prediction database yielded 269 potential targets, while disease target screening identified 1491 targets. Analysis using Venny2.1.0 revealed 97 intersection targets between drug and disease targets (Figure 4A). STRING database analysis at a confidence level of 0.4 generated a PPI interaction network comprising 97 nodes and 1075 edges (Figure 4B). Cytoscape 3.10.1 analysis identified the top eight core targets based on the Degree value: interleukin-6 (IL-6), serine/threonine protein kinase (AKT1), steroid receptor coactivator (SRC), prostaglandin endoperoxide synthase 2 (PTGS2), matrix metalloproteinase 9 (MMP9), peroxisome proliferator-activated receptor γ (PPARG), epidermal growth factor receptor (EGFR), and mitogen-activated protein kinase 3 (MAPK3) (Figure 4C,D).

GO and KEGG pathway enrichment analyses were performed using the DAVID platform. GO enrichment analysis revealed that PSE affected biological processes (BP) including signal transduction, positive regulation of transcription from RNA polymerase II promoter, and protein phosphorylation; cellular components (CC) including plasma membrane, nucleus, and cytoplasm; and molecular functions (MF) including protein binding, protein serine/threonine/tyrosine kinase activity, and enzyme binding (Figure 4E). KEGG analysis identified significant pathways including the PI3K-Akt signaling pathway, MAPK signaling pathway, and TNF signaling pathway (Figure 4F).

### 2.3. Transcriptomics Analysis of the DEGs

**Figure 5:**
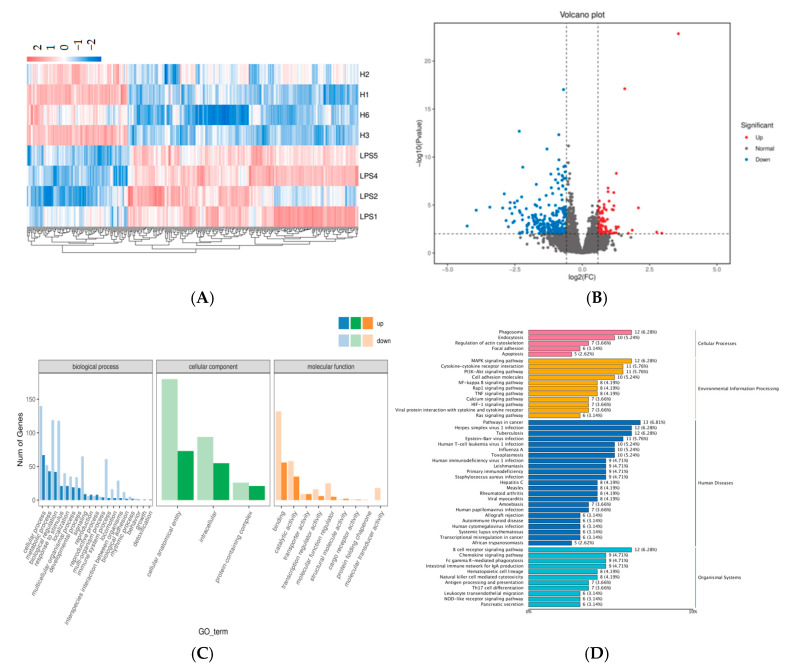
A cluster heat map showing the distribution of differential gene expression. Each group contains four biological replicates (**A**). A volcano map showing all DEGs; red, blue, and gray indicate that DEGs are significantly up-regulated, down-regulated, and non-significant, respectively (**B**). GO annotation of DEGs. The most significantly enriched parts of biological processes (BP), cellular components (CC), and molecular functions (MF) (**C**). Significantly enriched KEGG pathways based on DEGs (**D**).

Transcriptome sequencing performed on the BMK Cloud platform identified 342 significantly differentially expressed genes (DEGs), including 104 upregulated and 238 downregulated genes. Hierarchical cluster analysis and volcano plot visualization were constructed to represent the differential gene expression patterns (Figure 5A,B). GO enrichment analysis revealed that PSE affected various biological processes, including cellular processes, metabolic processes, biological regulation, and response to stimulus (Figure 5C). KEGG pathway analysis indicated that PSE modulated the MAPK, PI3K-AKT, and TNF pathways (Figure 5D).

### 2.4. The Effect of PSE on PI3K/AKT1 Pathway Protein in Intestinal Tissue

**Figure 6:**
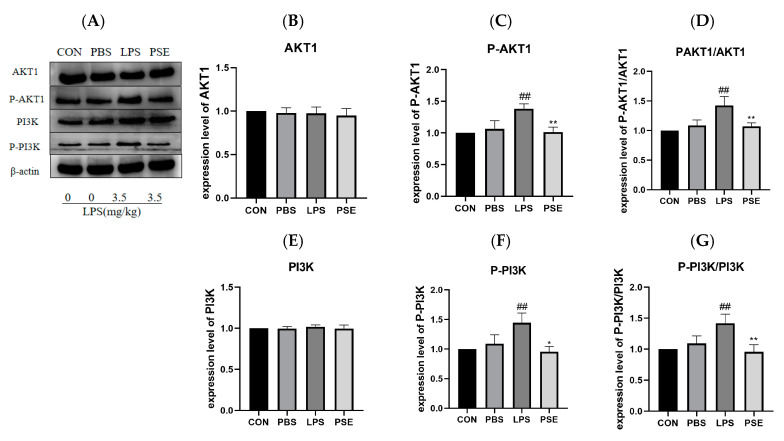
The effect of PSE on PI3K/AKT1 pathway protein in intestinal tissue. The experimental groups were divided into four groups: (i) CON (drinking water), (ii) PBS (PBS), (iii) LPS (3.5 mg/kg BW), (iv) PSE (3.5 mg/kg and PSE 100 mg/kg/d) *n* = 3. Representative Western blot images (**A**) and quantification of AKT1 (**B**), p-AKT1 (**C**), p-AKT1/AKT1 (**D**), PI3K (**E**), p-PI3K (**F**), p-PI3K/PI3K (**G**) protein expression. PSE inhibits PI3K and AKT1 phosphorylation. Data are expressed as mean ± SEM. ## *p* < 0.01 vs. control group; * *p* < 0.05, ** *p* < 0.01 vs. LPS control group.

The PI3K/AKT pathway was identified as significantly enriched in both network pharmacology and transcriptomics analyses. LPS treatment significantly increased the expression of p-AKT1 and p-PI3K compared to the CON group. PSE treatment significantly decreased the expression of both p-AKT1 and p-PI3K compared to the LPS group (Figure 6), indicating that PSE inhibited PI3K and AKT1 phosphorylation.

### 2.5. In Vitro Validation Results

#### 2.5.1. The Effects of PSE on LPS-Induced Inflammation, Oxidation, and Tight Junction–Related Factors in MODE-K Cells

**Figure 7:**
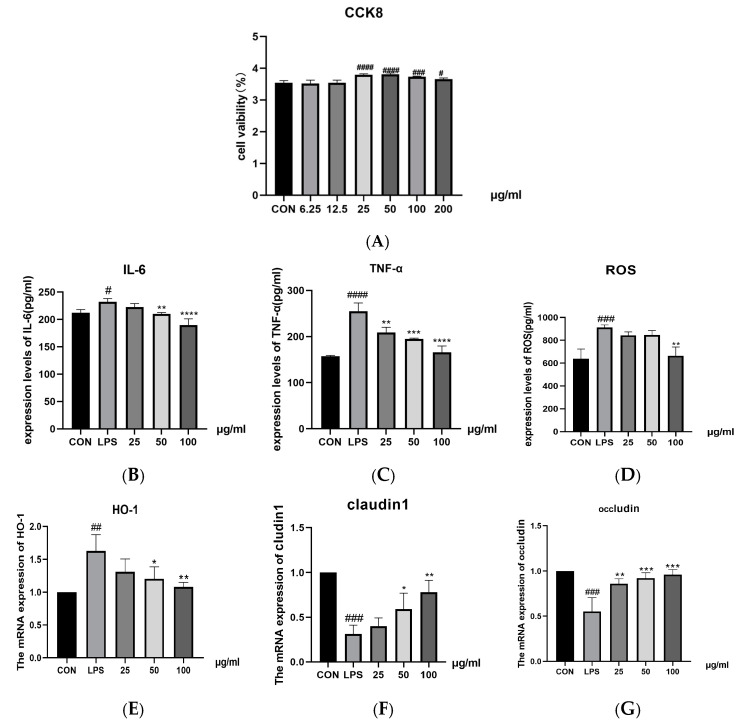
The effects of PSE on the regulation of inflammatory factors, oxidative indexes, and tight junctionfactors in LPS-induced MODE-K cells. MODE-K cells were divided into five groups: (i) CON (untreated), (ii) LPS (LPS (50 µg/mL) for 2 h), (iii) 25 (pretreatment with PSE (25 µg/mL) for 24 h + LPS (50 µg/mL) for 2 h), (iv) 50 (pretreatment with PSE (50 µg/mL) for 24 h + LPS (50 µg/mL) for 2 h), (v) 100 (pretreatment with PSE (100 µg/mL) for 24 h + LPS (50 µg/mL) for 2 h). *n* = 3. The effect of PSE on the viability of MODE-K cells (**A**). Inflammatory factors IL-6 and TNF-α (**B**,**C**), oxidative indicators ROS and HO-1 (**D**,**E**), tight junction factors Occludin and Claudin1 (**F**,**G**). PSE effectively inhibited the expression of inflammatory and oxidative factors in MODE-K cells and increased the expression of tight junction factors. Data are expressed as mean ± SEM. # *p* < 0.05, ## *p* < 0.01, ### *p* < 0.001, and #### *p* < 0.0001 vs. control cells; * *p* < 0.05, ** *p* < 0.01, *** *p* < 0.001, and **** *p* < 0.0001 vs. LPS-induced cells.

PSE treatment at concentrations of 25–200 μg/mL significantly increased cell viability compared to the CON group (*p* < 0.001, Figure 7A). Based on these results, concentrations of 25, 50, and 100 μg/mL PSE were selected for subsequent experiments. LPS treatment significantly increased inflammatory factors IL-6 (*p* < 0.05, Figure 7B) and TNF-α (*p* < 0.001, Figure 7C), oxidative stress markers ROS (*p* < 0.001, Figure 7D), and HO-1 mRNA expression (*p* < 0.01, Figure 7E), while decreasing mRNA expression of tight junction proteins Claudin1 and Occludin (*p* < 0.001, Figure 7F,G) compared to the CON group. PSE treatment significantly reversed these LPS-induced changes.

#### 2.5.2. The Effect of PSE on LPS-Induced PI3K/AKT1 Pathway Proteins in MODE-K Cells

**Figure 8:**
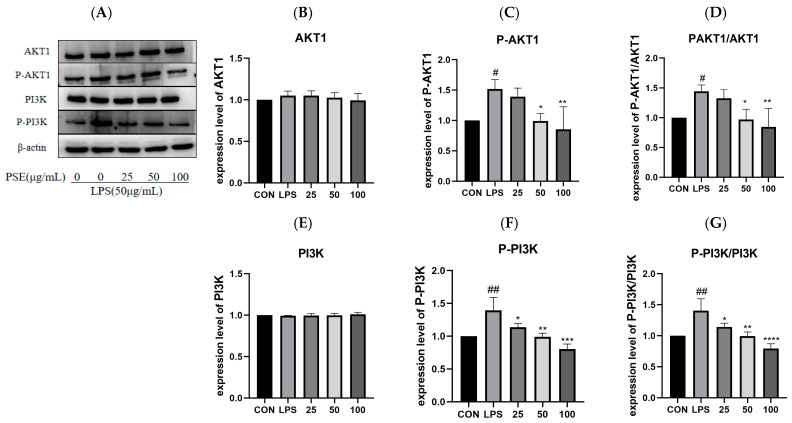
The effect of PSE on PI3K/Akt1 signaling pathway proteins in MODE-K cells. MODE-K cells were divided into five groups: (i) CON (untreated), (ii) LPS (LPS (50 µg/mL) for 2 h), (iii) 25 (pretreatment with PSE (25 µg/mL) for 24 h + LPS (50 µg/mL) for 2 h), (iv) 50 (pretreatment with PSE (50 µg/mL) for 24 h + LPS (50 µg/mL) for 2 h), (v) 100(pretreatment with PSE (100 µg/mL) for 24 h + LPS (50 µg/mL) for 2 h). *n* = 3. Representative Western blot images (**A**) and quantification of AKT1 (**B**), p-AKT1 (**C**), p-AKT1/AKT1 (**D**), PI3K (**E**), p-PI3K (**F**), p-PI3K/PI3K (**G**) protein expression. PSE inhibits PI3K and AKT1 phosphorylation. PSE can exert anti-inflammatory effects by inhibiting the phosphorylation of PI3K and AKT1 in the MODE-K cell inflammatory model. Data are expressed as mean ± SEM. # *p* < 0.05, ## *p* < 0.01, vs. control cells; * *p* < 0.05, ** *p* < 0.01, *** *p* < 0.001, and **** *p* < 0.0001 vs. LPS-induced cells.

Verification of PSE’s effects on PI3K/AKT1 pathway proteins was conducted at the cellular level (Figure 8A). LPS treatment significantly increased the phosphorylation levels of both AKT1 and PI3K compared to the CON group. PSE treatment at concentrations of 25–100 μg/mL significantly decreased the PI3K phosphorylation and p-PI3K/PI3K ratio, while PSE at 50 and 100 μg/mL significantly reduced the AKT1 phosphorylation and p-AKT1/AKT1 ratio (Figure 8D,G).

### 2.6. Component Analysis of PSE

**Figure 9:**
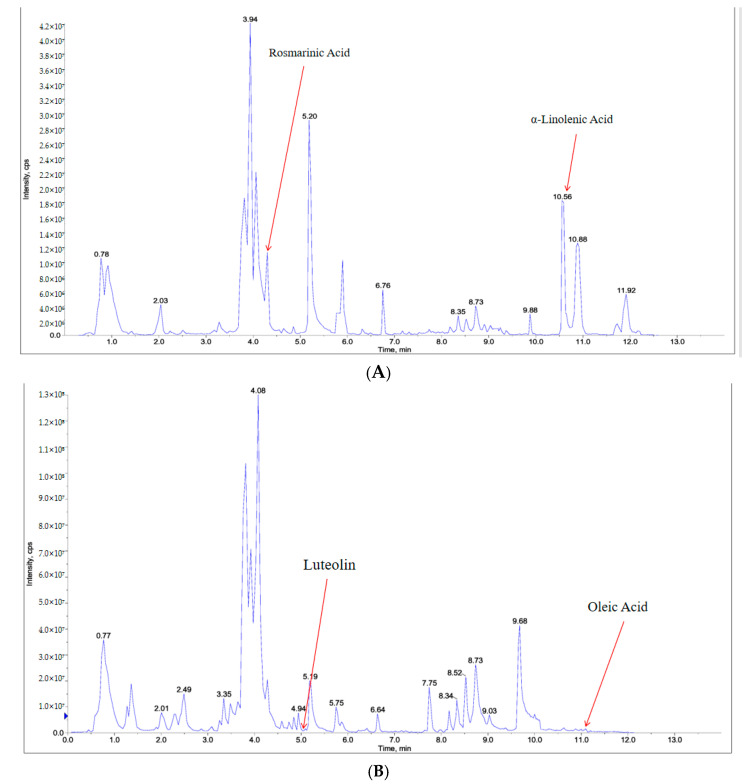
UPLC-MS/MS analysis of the chemical components of PSE. Chromatogram of the positive ion mode (**A**); chromatogram of the negative ion mode. The abscissa shows the retention time (Rt) of the metabolites, and the ordinate shows the ion current intensity, intensity unit: count per second (cps) (**B**).

Ultra-performance liquid chromatography–tandem mass spectrometry (UPLC-MS/MS) analysis was employed to identify the chemical constituents of PSE. The total ion current (TIC) chromatogram of the mixed quality control sample represents the intensity and time of all ions in the mass spectrum at each time point (Figure 9). A total of 1493 compounds were identified in PSE, comprising 393 flavonoids, 239 lipids, 190 amino acids and derivatives, 130 alkaloids, 119 phenolic acids, 79 terpenes, 59 organic acids, 43 lignans and coumarins, 43 nucleotides and derivatives, and 198 other compounds (Schedule 1). Among 16 active ingredients collected from TCMSP, 6 compounds met screening criteria: (E)-(4-methylbenzylidene)-(4-phenyltriazol-1-yl)amine, arachidonic acid, luteolin (content 0.41%), rosmarinic acid (content 0.27%), ALA (content 1.2%), and oleic acid (content 0.2%) (Table 1).

### 2.7. Molecular Docking Verification

**Figure 10:**
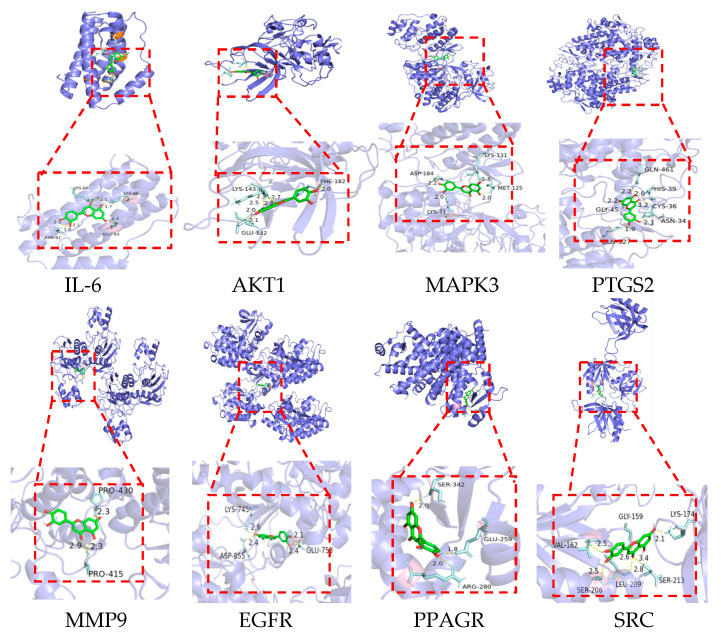
A schematic diagram of the optimal conformational interaction of Luteolin and core target docking. The PDB ID corresponding to the core target is: IL-6 (1ALU), AKT1 (3OS5), MAPK3 (4QTB), PTGS2 (5F19), MMP9 (5TH6), EGFR (7JXQ), PPAGR (8B8W), SRC (1U5D).

The eight core targets identified in Section 2.2 were used as receptors for molecular docking analysis with the six active ingredients screened in Section 2.6. The results are presented in Table 2. Luteolin demonstrated binding free energy values less than −5 kcal/mol with all eight targets, indicating stable binding potential with all targets. (E)-(4-methylbenzylidene)-(4-phenyltriazol-1-yl)amine exhibited stable binding with seven targets. Arachidonic acid showed stable interactions specifically with PTGS2 and PPARG. Rosmarinic acid demonstrated stable binding with PTGS2, while ALA formed stable bonds with PPARG. The visual representations of luteolin docking with core targets are illustrated in Figure 10.

### 2.8. The Effect of Luteolin on AKT1 Protein

**Figure 11:**
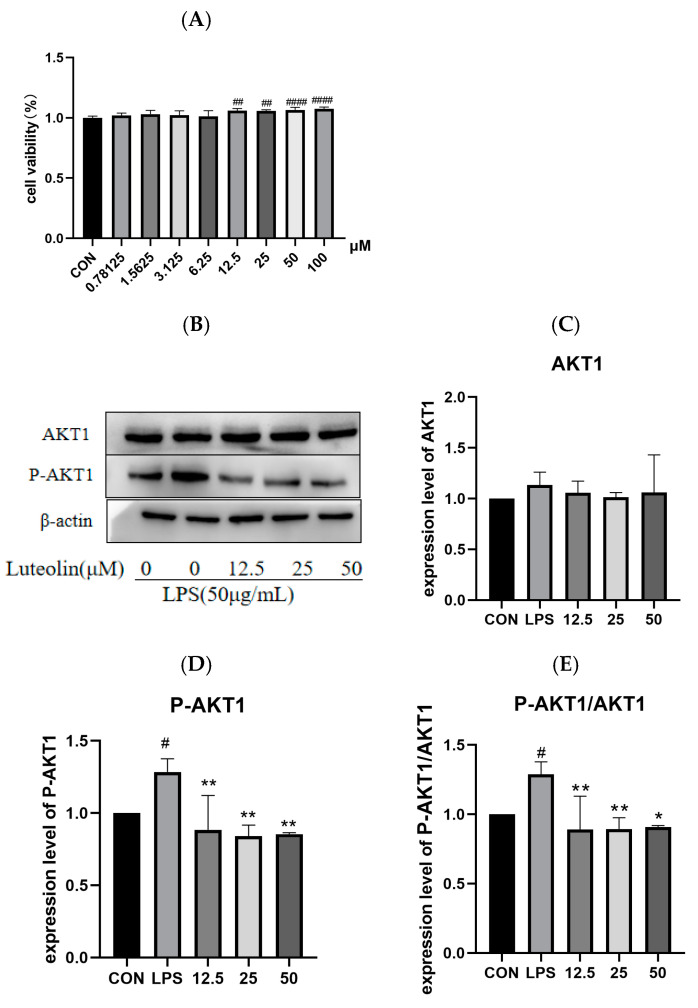
Effect of luteolin on AKT1 protein in LPS-induced MODE-K cells. MODE-K cells were divided into five groups: (i) CON (untreated), (ii) LPS (LPS (50 µg/mL) for 2 h), (iii) 12.5 (pretreatment with luteolin (12.5 μM) for 24 h + LPS (50 µg/mL) for 2 h), (iv) 50 (pretreatment with luteolin (50 μM) for 24 h + LPS (50 µg/mL) for 2 h), (v) 100 (pretreatment with luteolin (100 μM) for 24 h + LPS (50 µg/mL) for 2 h). *n* = 3. The effect of luteolin on the viability of MODE-K cells (**A**). Representative Western blot images (**B**) and quantification of AKT1 (**C**), p-AKT1 (**D**), p-AKT1/AKT1 (**E**) protein expression. Luteolin inhibits the phosphorylation of AKT1 protein in the MODE-K cell inflammatory model. Data are expressed as mean ± SEM. # *p* < 0.05 ## *p* < 0.01, and #### *p* < 0.0001vs.control cells; * *p* < 0.05, ** *p* < 0.01 vs. LPS-induced cells.

Cell viability was significantly increased following exposure to luteolin at concentrations of 12.5 μM, 25 μM, and 50 μM compared to the control group (*p* < 0.001, Figure 11A). These concentrations were selected for subsequent experiments. The LPS group showed significantly increased expression of the p-AKT1 and p-AKT1/AKT1 ratio compared to the control group (*p* < 0.05, Figure 11D,E). Luteolin treatment significantly decreased both p-AKT1 expression and the p-AKT1/AKT1 ratio compared to the LPS group (*p* < 0.01, Figure 11D,E).

## 3. Discussion

Inflammatory bowel disease (IBD), an idiopathic inflammatory disease of the intestines [18], is a multifactorial condition influenced by genetics, environment, diet, intestinal barrier function, and immune response [19,20]. Intestinal mucosal barrier dysfunction is considered a key factor in IBD pathogenesis [21]. Lipopolysaccharide (LPS), an integral component of Gram-negative bacterial cell membranes, has been demonstrated to activate inflammatory responses and compromise gastrointestinal barrier function [22,23,24,25,26,27,28]. Research has shown that LPS causes significant damage to intestinal morphology in vivo, reduces mRNA expression of tight junction proteins (Occludin and Claudin1), increases pro-inflammatory cytokine expression, and elevates reactive oxygen species (ROS) content both in vivo and in vitro, validating the IBD model establishment. Plant extracts have emerged as promising therapeutic options for IBD [29]. Previous research demonstrated that perilla extract effectively treated mice with colitis at doses ranging from 20–200 mg/kg, with 100 mg/kg identified as optimal [16,30]. The present study investigated the impact of perilla seed extract (PSE) treatment at 100 mg/kg on intestinal structure, demonstrating significant effects. Both in vivo and in vitro experiments showed that PSE increased mRNA expression of Occludin and Claudin1 while reducing oxidative stress and cytokine expression, suggesting PSE’s efficacy in treating IBD. Blood indicators, including eosinophil percentage (EOS%), neutrophil percentage (NEU%), and monocyte percentage (MON%), are characteristic of systemic inflammatory infection [31]. LPS treatment induced systemic inflammation in vivo, while PSE significantly reduced these inflammatory indices, potentially explaining the observed increases in body weight and food intake in PSE-treated mice. In our previous research, pretreatment with both *Siraitia grosvenorii* extract (SGE, 50–200 mg/kg BW) and baicalin methyl ester (BNE, 50–200 mg/kgBW) alleviated intestinal and systemic inflammation in the LPS-induced enteritis model of mice [22,32]. The recommended dose of PSE fell into their dose ranges, showing that the anti-inflammatory activities of PSE, SGE, and BNE are analogous.

Network pharmacology identified 97 shared drug-disease targets as potential mechanisms for PSE in IBD prevention. A protein–protein interaction (PPI) network analysis identified IL-6, AKT1, PTGS2, MMP9, PPARG, EGFR, and MAPK3 as core targets. Gene Ontology (GO) and Kyoto Encyclopedia of Genes and Genomes (KEGG) enrichment analyses identified 136 signaling pathways, primarily involving PI3K-AKT, MAPK, and tumor necrosis factor (TNF) signaling pathways. However, it should be noted that the application of a solitary network pharmacology analysis is constrained and may result in erroneous positive outcomes [33]. To circumvent the limitations of network pharmacology and enhance the reliability of the prediction, transcriptomics analysis was employed. The results of this analysis indicated an enrichment of the PI3K-AKT, MAPK, and TNF signaling pathways, which may be considered as potential specific targets and pathways for the treatment of IBD by PSE. The PI3K-AKT signaling pathway, a classical intracellular pathway responding to extracellular signals, regulates cell death, survival, and proliferation [34,35,36]. It plays a crucial role in intestinal homeostasis and has been identified as a key therapeutic target in IBD [37]. The MAPK signaling pathway, a fundamental inflammatory pathway, regulates cytokine synthesis and release during inflammatory responses and significantly influences intestinal inflammation [38,39]. The TNF signaling pathway, mediated by TNF, has been shown to initiate chronic inflammation and cell death in the intestine, with TNF inhibition representing a therapeutic approach to IBD [40,41]. AKT1, a core target involved in the PI3K-AKT signaling pathway, can treat IBD through cytokine inhibition via protein phosphorylation [42]. PI3K-AKT signal transduction rapidly activates downstream TNF and MAPK pathways [43,44]. Our research identified the PI3K-AKT signaling pathway as a pivotal target for PSE in IBD treatment. PSE was found to inhibit PI3K and AKT1 protein phosphorylation, suggesting its therapeutic mechanism in IBD. Other plant compounds, including celastrol and astragaloside IV, have also targeted the PI3K-AKT signaling pathway in treating ulcerative colitis [45,46].

UPLC-MS/MS analysis and network pharmacology prediction identified key chemical constituents in PSE, including luteolin, rosmarinic acid, ALA, and oleic acid. ALA, an Omega-3 fatty acid, has been well known to regulate the inflammatory response and can prevent DSS-induced colitis [30]. The UPLC-MS/MS analysis showed that the luteolin content of PSE was 1.52 times that of rosmarinic acid. Luteolin and rosmarinic acid demonstrated remarkable efficacy in mitigating dextran sulfate sodium (DSS)-induced colitis symptoms in mice at comparable dosages (luteolin: 25–50 mg/kg/day for 14 days; rosmarinic acid: 30–60 mg/kg/day for 7 days) [16,47,48,49]. However, significant differences exist at the cellular level. Luteolin reduced proinflammatory cytokine production in LPS-induced RAW264.7 cells at 0.29–0.58 μg/mL (1–2 μM, molecular weight = 286) [48,49], while rosmarinic acid alleviated *Salmonella enteritidis*–induced inflammation in RAW264.7 cells at 15–60 μg/mL [50], suggesting a significantly lower minimum effective dose for luteolin. It is hypothesized that luteolin is an important active substance in the PSE treating LPS-induced inflammation, based on a comparison of their content and efficacy.Binding free energy, a metric for evaluating component-target binding ability, indicates stronger affinities at lower values, with energies below −5 kcal/mol suggesting good affinity [51]. Luteolin demonstrated superior binding affinities to core target proteins compared to other constituents, with all binding free energies below −5 kcal/mol, identifying it as a potential marker for PSE in IBD treatment. In this manuscript, PSE (50–100 μg/mL) and luteolin (12.5–50 μM; 3.625–14.5 μg/mL) significantly reduced LPS-induced AKT1 protein phosphorylation in MODE-K cells. Luteolin has been demonstrated to inhibit LPS-induced inflammatory responses through modulation of PI3K-Akt signaling in RAW 264.7 cells [52], and it can also reduce hypertension via inhibition of the PI3K/Akt signaling pathway in the hypothalamus [53]. Furthermore, molecular dynamics simulation analysis demonstrates that luteolin exhibits strong binding affinity and stable interaction with AKT1 [54]. Consequently, the inhibition of luteolin-mediated AKT1 phosphorylation may represent a critical mechanism in PSE’s therapeutic effect on IBD.

The efficacy and safety of PSE can be referred to the PSO. In Wistar rats, the acute toxicity test showed that 20 g/kg PSO did not cause significant treatment-associated toxicity, and the sub-chronic toxicological evaluation of PSO (90-day toxicity test with a recovery period of 30 days) revealed that the ‘no-observed adverse effect level’ was determined to be 4 g/kg [55,56]. Luteolin alsohas a favorable safety profile when administered over an extended period. In the cystinosis model, treatment of mice with luteolin at a dose of 150 mg/kg/day for a period of 2–8 months did not result in the occurrence of any significant side effects [57].While this study provides scientific evidence for PSE’s efficacy in IBD treatment, certain limitations exist. Network pharmacology and transcriptomics analyses identified additional signaling pathways, suggesting that PSE may act through multiple targets and pathways. Moreover, the investigation focused primarily on luteolin’s effect on AKT1 protein, without exploring other components and targets. Further research is warranted to address these limitations.

## 4. Materials and Methods

### 4.1. Reagents

PSE was procured from Sichuan Hengrui Tongda Biotechnology Co., Ltd., (Dujiangyan, China) (Lot number: 240301). The following methodology was employed in the preparation of PSE. Firstly, 65–75% ethanol was added to the dried perilla seed powder, with a material-to-liquid ratio of 1:8. Secondly, the material was extracted by heating and refluxing for 2 h. Thirdly, the extraction process was repeated three times. Finally, the ethanol was recovered, concentrated, and spray-dried. PSE was adhered to the National Standard General Requirements for Natural Plant Feed Ingredients of the People’s Republic of China (GB/T 19424-2018). Mouse IL-6, TNF-α, and ROS assay kits were supplied by Jiangsu Enzyme Immunoassay Industry Co., Ltd. (Nanjing, China). LPS was purchased from Sigma Aldrich (St. Louis, MO, USA). Fetal bovine serum (FBS) was obtained from ZETA LIFE (Paris, France), while penicillin, streptomycin, and trypsin were acquired from GIBCO (Los Angeles, CA, USA). DMEM high-glucose medium was sourced from local biological companies. Antibodies in the experiments included Akt1 (1:1000, AF0836), P-Akt1 (Ser473) (1:1000, AF8355), PI3K p85 alpha (1:1000, AF6241), and P-PI3K p85 (Tyr458)/p55 (Tyr199) (1:1000, AF3242) from Affinity Bioscience Ltd., (Cincinnati, OH, USA). Anti-β-actin (1:1000, #R10602), horseradish peroxidase (HRP)–labeled goat anti-mouse IgG (1:1000, #R20619), and goat anti-rabbit IgG (1:1000, #R10327) antibodies were purchased from TransGen Biotechnology Co., Ltd., (Beijing, China).

### 4.2. Animal Experiment

Thirty-two male C57BL/6 mice (4–5 weeks old) were procured from Guangzhou Furuoge Biotechnology Co., Ltd. (Guangzhou, China). All experimental procedures were conducted in accordance with institutional guidelines for animal welfare and ethics. The mice were maintained under specific pathogen-free conditions in a controlled environment: ambient temperature of 26 ± 1 °C, a 12 h light/dark cycle, and free access to standard rodent chow and tap water. Upon arrival, the mice were housed in cages (3–4 per cage) and allowed a 7-day adaptation period. Subsequently, they were randomly allocated into four experimental groups (*n* = 8 per group): control group (CON), PBS group (intragastric (i.g.) administration of PBS; PBS), LPS group (normal feeding, LPS ip at 3.5mg/kg BW on day 14), and PSE group (100 mg/kg i.g. daily + LPS ip at 3.5mg/kg BW on day 14). Both LPS and PSE were dissolved in PBS and subsequently administered to mice. Mice were euthanized 24 h after LPS administration. Serum and jejunal tissues were collected for subsequent analyses. The hearts, livers, and lungs were collected for the purpose of calculating the organ index. All animal care and experimental procedures adhered to protocols approved by the Animal Care and Use Committee of Guangdong Ocean University School of Medicine (Approval Number: 2022-scuec-021) and complied with the Guidelines on Animal Welfare of Guangdong Ocean University School of Medicine.

### 4.3. Cell Culture and Model Establishment

MODE-K cells (mouse intestinal epithelial origin) were obtained from the BeNa Culture Collection (Beijing, China). Cells were cultured in DMEM supplemented with 10% FBS and 1% penicillin/streptomycin in a humidified incubator at 37 °C with 5% CO_2_. Cell viability was assessed using the Cell Counting Kit-8 (CCK-8) (CK001, Beijing HuameiShengke, Haidian, Beijing, China). MODE-K cells were treated with varying concentrations of PSE (0, 6.25, 12.5, 25, 50, 100, and 200 μg/mL) for 24 h, followed by the addition of 10 μL CCK-8 solution and incubation for 1 h. Absorbance was measured at 450 nm using a microplate reader(BioTek, Winooski, VT, USA). Similarly, the viability of MODE-K cells exposed to luteolin (0.78125 to 100 μM) was evaluated. MODE-K cells were divided into five groups: control group (CON), LPS group (50 μg/mL LPS for 2 h), and PSE group (low dose, 25 μg/mL PSE + LPS; middle dose, 50 μg/mL PSE + LPS; high dose, 100 μg/mL PSE + LPS). The LPS concentration for cell experiments (50 μg/mL, 2 h) was referenced from previous laboratory studies [22]. PSE treatment was prepared as follows: 1 g PSE was dissolved in 1 mL of dimethyl sulfoxide (DMSO), then the solution was filtered through a 0.22 μm bacterial filter to obtain the PSE stock solution. Before adding PSE to the cells, the PSE stock solution was diluted with culture medium to the concentration of 25–100 μg/mL. The luteolin treatment in MODE-K cells (12.5 μM, 25 μM, and 50 μM) was the same as that for PSE.

### 4.4. HE and PAS Staining

Jejunal tissues from mice were fixed in 4% paraformaldehyde, embedded in paraffin, sectioned, and stained with hematoxylin-eosin (HE) and periodic acid–Schiff (PAS) stain. Histopathological alterations, including villus length, crypt depth, and goblet cell counts, were evaluated using an optical microscope (Olympus, Tokyo, Japan) and Image Pro Plus 6.0 software(Media Cybernetics, Rockville, MD, USA).

### 4.5. RNA Extraction, Reverse Transcription, and Real-Time PCR

RNA was extracted from jejunal epithelial cells using TransZol Up (TransGen Biotechnology, Beijing, China) according to the manufacturer’s protocol. RNA concentration and purity were determined using a NanoDrop 2000 spectrophotometer (Thermo Fisher Scientific, Waltham, MA, USA). Complementary DNA (cDNA) synthesis was performed using HifairIII 1st Strand cDNA Synthesis SuperMix for qPCR (Yeasen Biotechnology, Shanghai, China). Real-time quantitative PCR was conducted using Hieff UNICON Universal Blue qPCR SYBR Green Master Mix (Yeasen Biotechnology, Shanghai, China) on a Fluorescent Quantitative PCR Detection System (FQD-96X, Hangzhou Bori Technology, Hangzhou, China). Table 3 displays the primer sequences that were synthesized by General Shanghai Sangong Bioengineering Technology Service Co., Ltd. (Shanghai, China). for PCR amplification. The relative expression of mRNA was computed, with *β-actin* as an internal control, using the 2^−ΔΔCT^ method.

### 4.6. Western Blotting

Proteins were extracted from MODE-K cells and jejunal tissues using RIPA lysis buffer (Beyotime, Beijing, China). Protein concentration was quantified using a BCA Protein Assay Kit (KeyGen, Nanjing, China). Equal amounts of protein were separated via SDS-PAGE and transferred onto PVDF membranes. Membranes were blocked, incubated overnight at 4 °C with primary antibodies (AKT1, P-AKT1, PI3K, P-PI3K, and β-actin; 1:1000 dilution), followed by incubation with HRP-conjugated secondary antibodies (1:1000) for 2 h. Protein bands were visualized using chemiluminescence and quantified using Image J software (v1.8.0).

### 4.7. ELISA

ELISA kits from Jiangsu Enzyme Immunoassay Industry Co., Ltd. (Nanjing, China) were utilized to quantify IL-6, TNF-α, and ROS concentrations in cells and jejunal tissues, following the manufacturer’s protocols. 

### 4.8. Network Pharmacology

#### 4.8.1. Screening of PSE for Active Components and Targets of Action

The Traditional Chinese Medicine Systems Pharmacology Database and Analysis Platform (TCMSP) was used to identify active components of PSE with oral bioavailability (OB) ≥ 30% and drug-likeness (DL) ≥ 0.18 as criteria. Additional components were referenced from the Chinese Pharmacopoeia, literature, and the SymMap database. SMILES data for the active ingredients were retrieved from the PubChem database, and their potential targets were identified using the SwissTargetPrediction platform.

#### 4.8.2. IBD Target Screening

The search term ‘inflammatory bowel disease’ was employed to identify disease targets associated with inflammatory bowel disease from the GeneCards (https://www.genecards.org/), DisGeNET (https://www.disgenet.org/), and OMIM (https://www.omim.org/) databases. In order to identify targets related to inflammatory bowel disease, it is necessary to organize the data set and remove any genes that are duplicated.

#### 4.8.3. Protein–Single-Bond Protein Interaction (PPI) Network Construction

Intersecting targets from PSE components and IBD-related targets were identified using the Venny 2.1.0 platform. The STRING database (minimum interaction score > 0.4) was used to construct the PPI network, which was visualized using Cytoscape 3.10.1 software. Network topology analysis via the CytoNCA plugin identified key core targets based on the selection criteria of the Degree, Betweenness Centrality (BC), and Closeness Centrality (CC) metrics.

#### 4.8.4. GO and KEGG Analysis

Gene Ontology (GO) and Kyoto Encyclopedia of Genes and Genomes (KEGG) pathway enrichment analyses were conducted using the DAVID database, with *p* < 0.05 as the threshold. The top 10 GO terms and top 20 KEGG pathways were visualized using the MicroBioinformatics platform.

### 4.9. Transcriptomics Study

RNA sequencing was performed by BioMac Biotechnology Co., Ltd. (Beijing, China), and data analysis was conducted using the BMKCloud platform (www.biocloud.net). Differentially expressed genes (DEGs) were identified using a corrected *p*-value < 0.05 as the threshold for significance.

### 4.10. UPLC-MS/MS Analysis

The chemical constituents of PSE were identified by UPLC-MS/MS analysis, which was entrusted to Wuhan Maiwei Metabolic Biotechnology Co., Ltd. (Wuhan, China). The specific experimental steps are as follows.

#### 4.10.1. Dry Sample Extraction

Using vacuum freeze-drying technology, the samples were placed in a lyophilizer (Scientz-100F)(Ningbo Xinzhi Biotechnology Co., Ltd., Ningbo, China), then ground (30 Hz, 1.5 min) to powder form by using a grinder (MM 400, Retsch) (Verder Shanghai Instruments and Equipment Co., Ltd., Shanghai, China). Next, 50 mg of the sample powder was weighed using an electronic balance (MS105DΜ) and 1200 μL of −20 °C pre-cooled 70% methanolic aqueous internal standard extract was added (less than 50 mg added at the rate of 1200 μL extractant per 50 mg sample). The sample was vortexed once every 30 min for 30 s, for a total of six times. After centrifugation (rotation speed 12,000 rpm, 3 min), the supernatant was aspirated, and the sample was filtered through a microporous membrane (0.22 μm pore size) and stored in the injection vial for UPLC-MS/MS analysis.

#### 4.10.2. UPLC Conditions

The sample extracts were analyzed using a UPLC-ESI-MS/MS system (UPLC, ExionLC™ AD, https://sciex.com.cn/) and a tandem mass spectrometry system (https://sciex.com.cn/). The analytical conditions were as follows. The UPLC system utilized an Agilent SB-C18 column (1.8 µm, 2.1 mm × 100 mm). The mobile phase consisted of solvent A, which was pure water with 0.1% formic acid, and solvent B, which was acetonitrile with 0.1% formic acid. Sample measurements were performed using a gradient program starting with 95% A and 5% B. Over 9 min, a linear gradient transitioned to 5% A and 95% B, which was maintained for 1 min. Subsequently, the composition was adjusted back to 95% A and 5% B over 1.1 min and held constant for an additional 2.9 min. The flow rate was set at 0.35 mL/min, the column oven temperature was maintained at 40 °C, and the injection volume was 2 µL. The effluent was alternately connected to an ESI-triple quadrupole-linear ion trap (QTRAP)-MS system.

#### 4.10.3. ESI-Q TRAP-MS/MS

The ESI source operation parameters were as follows. The source temperature was set at 500 °C, with ion spray voltages of 5500 V for positive ion mode and −4500 V for negative ion mode. The ion source gas I (GSI), gas II (GSII), and curtain gas (CUR) were maintained at 50, 60, and 25 psi, respectively, while the collision-activated dissociation (CAD) was set to high. QQQ scans were performed using multiple reaction monitoring (MRM) experiments with nitrogen as the collision gas, set to medium. The declustering potential (DP) and collision energy (CE) for each MRM transition were further optimized, and specific MRM transitions were monitored based on the metabolites eluted during each period.

### 4.11. Molecular Docking

The molecular docking process began by identifying the active ingredient in TCMSP using its chemical name and downloading its structural file in mol2 format. The mol2 file was imported into AutodockTools software (Version 1.5.7) for hydrogenation, torsion bond setting, and other ligand preparation steps, after which it was saved in pdbqt format. For receptor preparation, protein structures were filtered from the PDB database using X-ray diffraction data with a resolution below 3 Å. The selected pdb file underwent modifications, including dewatering, hydrogenation, and receptor setting, all performed using PyMol (Version 1.8.6) and AutodockTools software. The processed file was then exported in pdbqt format. The docking process required selecting the Grid option in AutodockTools and importing the pdbqt files of the receptor and ligand. Semi-flexible docking was configured, and GridBox was used to contain the receptor. The resulting configuration was saved in gpf format. The AutoGrid program was executed with the genetic algorithm parameter set to 50, while all other parameters remained at default. Docking results were saved in dpf format, and Autodock was run to identify the conformation with the best binding energy, which was saved in pdbqt format. Visualization of the results was performed using PyMol.

### 4.12. Statistical Analyses

All results were expressed as mean ± standard deviation (SD) values derived from three independent experiments. Statistical analyses were conducted using GraphPad Prism 9.0.2 software. Differences between groups were assessed using a *t*-test for comparisons between two groups or one-way ANOVA for comparisons among multiple groups. The findings were reported as ‘mean ± SD’, with a significance level of *p* < 0.05 indicating statistical significance and *p* < 0.01 indicating high statistical significance. Normality tests were conducted before applying parametric tests.

## 5. Conclusions

**Figure 12:**
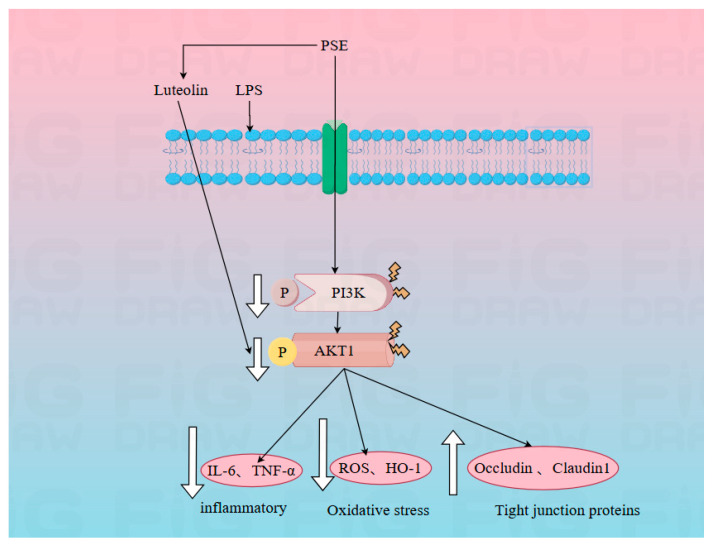
PSE and luteolin protect against inflammatory bowel disease model via the PI3K/AKT signal pathway. Note: The white arrow indicates the upregulation or downregulation of key indicators by PSE during the inflammatory process.

PSE demonstrates efficacy against IBD progression by enhancing intestinal barrier function and inhibiting inflammatory responses and oxidative stress via the PI3K/AKT1 signaling pathway. Luteolin’s inhibition of AKT1 protein phosphorylation appears to play a particularly crucial role in this therapeutic mechanism (Figure 12).

## Figures and Tables

**Table 1:** Six compounds of PSE.

MOL ID	Name	Formula	OB%	DL
MOL012893	(E)-(4-methylbenzylidene)-(4-phenyltriazol-1-yl)amine	C_16_H_14_N_4_	57.87	0.19
MOL001439	arachidonic acid	C_20_H_32_O_2_	45.57	0.2
MOL000006	Luteolin	C_15_H_10_O_6_	36.16	0.25
MOL011865	Rosmarinic Acid	C_18_H_16_O_8_	1.38	0.35
MOL000432	α-Linolenic Acid	C_18_H_30_O_2_	45.01	1.14
MOL000675	Oleic Acid	C_18_H_34_O_2_	33.13	0.14

**Table 2:** Molecular docking binding free energy results of perilla seed extract and inflammatory bowel disease.

Target	IL-6	AKT1	SRC	PTGS2	MMP9	PPARG	EGFR	MAPK3
Name								
Rosmarinic Acid	−4.42	−5.22	−3.07	−6.29	−4.93	−4.29	−4.38	−5.82
Luteolin	−5.87	−6.96	−5.2	−7.46	−6.59	−5.47	−6.81	−6.9
alpha-Linolenic Acid	−4.5	−4.7	−2.76	−4.8	−4.07	−5.12	−5.83	−4.29
Oleic Acid	−3.6	−4.87	−1.82	−4.44	−4.7	−4.29	−5.9	−4.95
Arachidonic acid	−4.3	−4.58	−1.5	−5.42	−4.15	−5.77	−4.99	−4.67
(E)-(4-methylbenzylidene)-(4-phenyltriazol-1-yl)amine	−6.69	−7.81	−4.68	−7.72	−9.73	−6.89	−8.37	−7.73

**Table 3:** Primers used for real-time PCR.

Gene	Primer Sequence (5′ to 3′)	Prodsize	Login Number
*β* *-actin*	F:GATCTGGCACCACACCTTCTACAAC	107	NC_010445.4
	R:TCATCTTCTCACGGTTGGCTTTGG		
*HO-1*	F:AAGCCGAGAATGCTGAGTTCA	106	NC_000074.7
	R:GCCGTGTAGATATGGTACAAGGA		
*Claudin1*	F:GCACAGCACTTTACAAGCAACC	117	NW_004624730.1
	R:TCGTCATCTTCCAAGCACTTCATAC		
*Occludin*	F:TGGCTATGGCGGATATACAGACC	86	NC_000079.7
	R:TTACTAAGGAAGCGATGAAGCAGAAG		

## Data Availability

The original data of the study are included in the Supplementary Material. Enquiries can be directed to the corresponding author.

## Supplementary Materials

The following supporting information can be downloaded at: https://www.mdpi.com/article/10.3390/ijms26083564/s1.

## Abbreviations

The following abbreviations are used in this manuscript:

PSE	Perilla seed extract
UPLC-MS/MS	Ultra-performance liquid chromatography–tandem mass spectrometry
TCMSP	Traditional Chinese medicine systems pharmacology
PPI	Protein–protein interaction
H&E	Hematoxylin and eosin
PAS	Periodic acid–Schiff stain
GO	Gene Ontology
KEGG	Kyoto Encyclopedia of Genes and Genomes
BP	Biological process
CC	Cellular component
MF	Molecular function
LPS	Lipopolysaccharide
OB	Oral bioavailability
DL	Drug-likeness
DEGs	Differentially expressed genes
