# Supplementary material for: Protect Effects of Perilla Seed Extract and Its Active Ingredient Luteolin Against Inflammatory Bowel Disease Model via the PI3K/AKT Signal Pathway In Vivo and In Vitro
Source: Int J Mol Sci. 2025 Apr 10;26(8):3564. doi: 10.3390/ijms26083564 (PMC12026851; doi:10.3390/ijms26083564)

1 Figure S1. The effect of PSE on PI3K/AKT1 pathway protein in intestinal tissue.

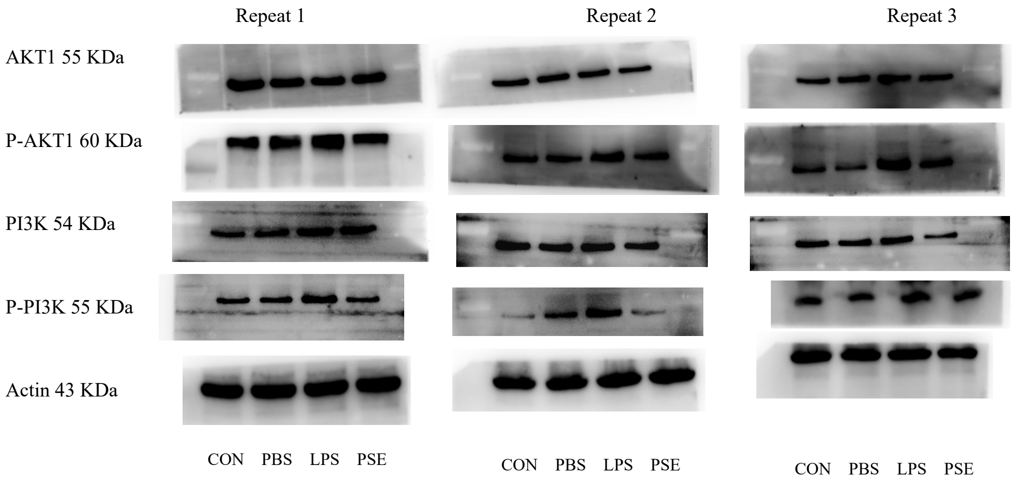

2 Figure S2. The effect of PSE on PI3K/Akt1 signaling pathway proteins in MODE-K cells.

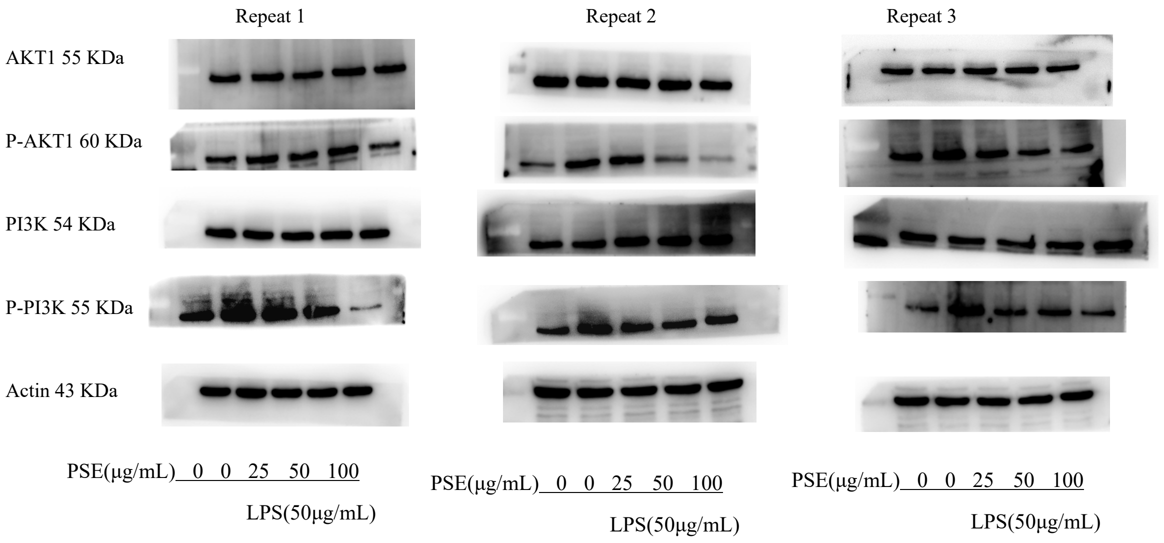

3 Figure S3. Effect of Luteolin on AKT1 protein in LPS-induced MODE-K cells.

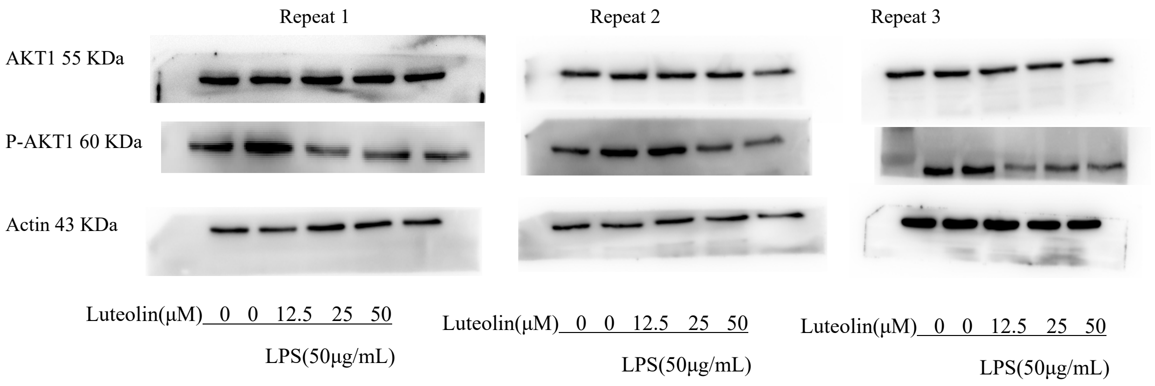

Supplement: Supplementary file 1 [file ijms-26-03564-s001.zip › ijms-3489319-supplementary.pdf]
